# Efficacy and safety of vornorexant in Japanese patients with insomnia: a randomized, placebo-controlled phase 3 pivotal study

**DOI:** 10.1093/sleep/zsaf291

**Published:** 2025-09-26

**Authors:** Makoto Uchiyama, Daiji Kambe, Sayaka Hasegawa, Yumiko Imadera, Hironori Yamasaki, Naohisa Uchimura

**Affiliations:** Tokyoadachi Hospital, Tokyo, Japan; Department of Psychiatry, Nihon University School of Medicine, Tokyo, Japan; Development Headquarters, Taisho Pharmaceutical Co., Ltd., Tokyo, Japan; Development Headquarters, Taisho Pharmaceutical Co., Ltd., Tokyo, Japan; Development Headquarters, Taisho Pharmaceutical Co., Ltd., Tokyo, Japan; Development Headquarters, Taisho Pharmaceutical Co., Ltd., Tokyo, Japan; Department of Neuropsychiatry, Kurume University School of Medicine, Fukuoka, Japan

**Keywords:** TS-142, ORN0829, orexin receptor antagonist, clinical trial, hypnotics, sleep, vornorexant, insomnia, placebo-controlled, phase 3

## Abstract

**Study Objectives:**

A placebo (PBO)-controlled phase 3 study was conducted to evaluate the efficacy and safety of vornorexant, a novel dual orexin receptor antagonist, in Japanese patients with insomnia.

**Methods:**

Following a 2-week PBO run-in period, 596 patients with insomnia were randomized to receive 5 mg of vornorexant (VOR5), 10 mg of vornorexant (VOR10), or PBO for 2 weeks in a 1:1:1 ratio in double-blind manner, followed by a 1-week PBO run-out period. The primary and key secondary efficacy endpoints were subjective sleep latency and subjective sleep efficiency, respectively, assessed by sleep diary.

**Results:**

Subjective sleep latency was significantly improved at week 2 in both groups compared to PBO (differences of the least-squares mean [LSM] changes from baseline in VOR5, −10.6 min [95% CI = −14.2% to −7.0%] *p* < .001, and VOR10, −10.1 min [−13.8% to −6.5%] *p* < .001). Subjective sleep efficiency was also significantly improved at 2 weeks in both groups compared to PBO (LSM changes from baseline in VOR5, 3.41% [1.87% to 4.96%] *p* < .001, and VOR10, 2.94% [1.40% to 4.48%] *p* < .001). The most common adverse event (AE) was somnolence (VOR5: 3.1%, VOR10: 3.6%, PBO: 1.5%). No AEs of cataplexy, fall, muscular weakness, sleep paralysis, hypnagogic or hypnopompic hallucination, or excessive daytime sleepiness were reported. No withdrawal symptoms or rebound insomnia were observed in the PBO run-out period.

**Conclusions:**

Treatment with 5 and 10 mg of vornorexant significantly improved sleep onset latency and sleep efficiency compared with PBO without relevant safety concerns.

**Clinical Trial Registration:**

ClinicalTrials.gov ID: NCT05453136. Japan Registry of Clinical Trials ID: jRCT2031220209. A Multi-center, Randomized, Double-blind, Parallel-group, Placebo-controlled Phase 3 Study of TS-142 in Patients with Insomnia Disorder. https://clinicaltrials.gov/study/NCT05453136. https://jrct.mhlw.go.jp/en-latest-detail/jRCT2031220209.

**Statement of Significance**

Sleep disturbances lead to physical and mental health issues. Some pharmacotherapies for individuals with insomnia are available, but most of these have concerns including withdrawal, dependence, and next-day residual effects. Therefore, alternative treatment with minimal impact on these issues is highly desirable. This phase 3 study was conducted to confirm the efficacy and safety of vornorexant, a novel dual orexin antagonist having pharmacokinetic characteristics of rapid absorption and elimination, in patients with insomnia. Consequently, both tested doses showed superior efficacy compared to placebo, with significant reductions in subjective sleep latency and increases in sleep efficiency. These findings position vornorexant as a new treatment option for insomnia, offering improvements in sleep while avoiding the concerns associated with traditional hypnotics.

## Graphical Abstract

**Graphical Abstract:**
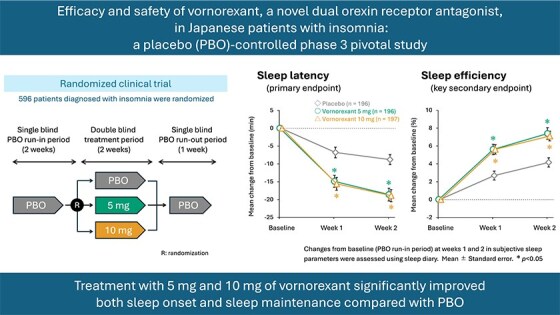

## Introduction

Insomnia is defined as some complaints of difficulty with sleep initiation, maintenance, duration, or consolidation, together with various daytime consequences [1]. Chronic insomnia disorder is currently characterized as the most prevalent sleep disorder [2]. Symptoms of insomnia are more likely to persist than remit after a time of stress or uncertainty [3]. Persistent sleep disturbances lead to subsequent physical and mental health problems [4–6], hence effective treatments are urgently needed. Insomnia treatment primarily involves two approaches: nonpharmacotherapy and pharmacotherapy. Each approach offers well-established options: cognitive behavioral therapy for insomnia (CBT-i) and pharmacologic treatments such as benzodiazepine receptor agonists (BzRAs), melatonin receptor agonists, histamine H_1_ receptor antagonists, and antidepressants [7]. These options prominently address sleep improvement. However, their limitations include an access barrier for CBT-i [8] and the development of dependence on BzRAs [9]. These concerns have warranted new treatment options that are more accessible and safer for physicians and patients with insomnia.

Orexin receptor type 1 (OX_1_) and type 2 (OX_2_) are G-protein coupled receptors which were described in the late 1990s [10]. The ligands for these receptors are neuropeptides called orexin-A and orexin-B, which are predominantly expressed in the brain. Genetic, molecular, and pharmacological studies have revealed that orexin signaling regulates the sleep/wake cycle in rodents, dogs, and humans. Potentiation of orexinergic signaling by transgene [11], optogenetic stimulation [12], and peptidic agonists [13, 14] induced significant wakefulness, whereas gene knockout [15], chemogenetic silencing [16], and various antagonists [17, 18] induced sleep. Moreover, the loss of orexinergic neurons or low levels of orexin-A in cerebrospinal fluid are strongly linked to narcolepsy, a chronic sleep disorder characterized by excessive daytime sleepiness, sleep attacks, and/or cataplexy [19, 20]. These findings have led to the development of new hypnotics with action against orexin receptors. Small molecules which antagonize both OX_1_ and OX_2_ (dual orexin receptor antagonists [DORAs]) have demonstrated significant efficacy for sleep difficulties and favorable safety characteristics which may avoid some of the concerns associated with BzRAs. However, commercially available DORAs have long half-lives (approximately 12 h for suvorexant [21], 17-19 h effective half-life for Lemborexant [22], and approximately 8 h for daridorexant [23]), which has raised concerns over residual effects.

Vornorexant (development code name: TS-142) is a novel DORA with high affinity and selectivity for both OX_1_ and OX_2_ [24]. It has been developed for the treatment of insomnia, with the aim of improving difficulties in sleep initiation and sleep maintenance, as well as daytime functioning and quality of life. Furthermore, to avoid the next-day residual effect, vornorexant has a unique pharmacokinetic characteristic: the shortest elimination half-life (1.32–3.25 h) among clinically available DORAs [25, 26]. An exploratory study revealed that this novel DORA ameliorated both objective sleep parameters (e.g., latency to persistent sleep [LPS], wake time after sleep onset [WASO], sleep efficiency [SE], total sleep time [TST]) assessed by polysomnography (PSG) and subjective measures (e.g., subjective sleep latency [sSL], subjective TST [sTST], subjective WASO [sWASO]) assessed by sleep questionnaire in patients with insomnia [27]. In this article, we report the results of a confirmatory phase 3 clinical trial that evaluated the efficacy and safety of vornorexant in Japanese patients with insomnia.

## Materials and Methods

This study was conducted as a multi-center, randomized, double-blind, placebo (PBO)-controlled phase 3 confirmatory study. The clinical study protocol and informed consent form for this trial were reviewed and approved by the Japanese regulatory agency and the institutional review boards of participating institutions prior to conducting the clinical study. No patients were involved in designing the study. The study was registered at ClinicalTrials.gov (https://clinicaltrials.gov/) under the identifier NCT05453136 on July 7, 2022 and at Japan Registry of Clinical Trials (jRCT: https://jrct.mhlw.go.jp/search?kanguage=en) under the identifier jRCT2031220209 on July 9, 2022. This clinical study was conducted by Taisho Pharmaceutical Co., Ltd. in accordance with the ethical principles stated in the Declaration of Helsinki, local regulatory laws, and the Good Clinical Practice (GCP) guidelines. Written informed consent was obtained from all patients prior to their enrollment in the study. Patients were reimbursed for their travel expenditure by the study sponsor. A full list of the clinical study sites is provided in Supplementary Material S1. In line with the Japanese guideline on the clinical evaluation of sleeping drugs [28], key aspects of the trial design—including the selection of sleep assessment tools such as PSG or sleep diaries, the definition of primary and key secondary endpoints, and the overall study duration—were agreed upon in consultation with the Japanese regulatory authorities prior to the initiation of the study. Study methods and results are reported following the Consolidated Standards of Reporting Trials 2025 statement for parallel-group randomized trials [29]. The clinical study protocol and the statistical analysis plan are available on the jRCT website at https://jrct.mhlw.go.jp/latest-detail/jRCT2031220209.

### Patient recruitment and selection

Male and female nonelderly (18–64 years) and elderly (65 years or more) outpatients who met the Diagnostic and Statistical Manual of Mental Disorders-Fifth Edition definition (DSM-5) for insomnia disorder were enrolled. Patients with a known history or clinical suspicion of other DSM-5-defined sleep–wake disorders—including breathing-related sleep disorder, circadian rhythm sleep–wake disorders, parasomnias, and others—were excluded. While formal diagnostic procedures such as PSG were not conducted for all participants, physicians carefully reviewed medical histories and conducted clinical assessments to exclude individuals with apparent symptoms suggestive of other sleep–wake disorders. Patients with psychiatric disorders other than sleep disorders or comorbidities causing severe sleep difficulties were excluded. Eligible patients were required to have a history of sleep difficulty measured daily using an electronic patient-reported outcome sleep diary, defined as sSL of 30 min or more and sTST of less than 6.5 h at least 4 nights in the last 7 nights of PBO run-in period. A sleep history of bedtime between 21:00 and 1:00 and time spent in bed for between 6.5 and 9 h at least 4 nights in the last 7 nights of the PBO run-in period was also required. Laboratory PSG screening was not conducted in this study. Patients with a STOP-Bang test score of 5 or more, indicating potential sleep apnea–hypopnea, were excluded [30]. Patients whose Insomnia Severity Index score exceeded 14 (moderate or severe insomnia) [31] were included, but those who improved by more than 5 points during the PBO run-in period were excluded [32]. A complete list of inclusion and exclusion criteria is shown in Supplementary Material S1.

### Study design

This study consisted of a 2-week single (patient only)-blind PBO run-in period and a 2-week double-blind treatment period, followed by a 1-week single (patient only)-blind PBO run-out period (Supplementary Material S1). At the last visits in the PBO run-in period (Visit 3), eligible patients were randomized to one of three parallel groups (vornorexant 5 mg [VOR5], vornorexant 10 mg [VOR10], or PBO; fixed dose) in a 1:1:1 ratio using an Interactive Web Response System. Randomization was stratified using a dynamic allocation method to balance across the factors of age (<65 or ≥65 years) and sSL at baseline (<40, ≥40 but <80, ≥80 but <120, ≥120 min). Treatment allocation was kept strictly confidential except for the allocation staff and the emergency/suspected unexpected serious adverse reaction key code managers in this clinical study until database lock. All investigational drugs and these packages were manufactured by Taisho pharmaceutical to be identical in appearance. The investigational drugs were administered orally once daily around 5 min prior to bedtime.

### Assessments of efficacy and safety endpoints

Sleep-related parameters such as sSL, subjective SE (sSE), sTST, sWASO, subjective number of awakenings (sNAW), and frequency of nighttime urination were assessed using the electronic sleep diary recorded through the clinical study periods (5 weeks). Sleep-related efficacy was also assessed using patient-reported measures (Leeds Sleep Evaluation Questionnaire [33] and Insomnia Severity Index [31]), as well as physician-reported measures (Clinical Global Impression-Improvement [CGI-I] and Clinical Global Impression-Severity [CGI-S] scales [34]). Items focusing on the patients’ daytime functioning were assessed using the Sheehan Disability Scale [35] alongside the sleep-related assessments, but some of these results will be reported separately.

Safety assessment included adverse events (AEs) including predefined grouped events of clinical interest (ECIs, defined in Supplementary Material S1), body weight, vital signs, electrocardiography, and laboratory tests. Additionally, the Karolinska Sleepiness Scale (KSS; for next-day residual effect) [36], Digit Symbol Substitution Test (DSST; for cognitive function) [37], Benzodiazepine Withdrawal Symptom Questionnaire (BWSQ) [38], and Colombia-Suicide Severity Rating Scale (C-SSRS) [39] were evaluated. Rebound insomnia was assessed using the sleep diary comparison between baseline values (PBO run-in period) and values during drug withdrawal (PBO run-out period). These efficacy and safety outcomes were predefined and have remained unchanged since study initiation, and the prespecified statistical analysis underwent no significant modifications from its original version.

### Statistical analysis

sSL and sSE were defined as the primary and key secondary endpoints, respectively. All other endpoints were set as secondary endpoints.

The primary analysis for the primary and key secondary endpoints was defined as the comparison between change from baseline on week 2 of VOR5 or VOR10 and PBO. All efficacy analyses were performed on the full analysis set which included all patients who received at least one dose of the investigational drug during the treatment period and had at least one post-treatment efficacy measurement. The changes from baseline values on the time frame (weeks 1 and 2) were summarized as arithmetic mean with standard error of the mean (SEM) as well as analyzed using a linear mixed-effects model for repeated measures (MMRM), with the factors of treatment, time frame, and interactions of treatment by time frame as fixed effects and the baseline value as a covariate. In this analysis, it was assumed that missing data were missing at random (MAR), and no imputation was performed. Results were reported as the point estimates of least-squares means (LSM) and their two-sided 95% confidence intervals (CIs).

The main null hypotheses were as follows: there was no difference between (1) VOR10 and PBO in sSL, (2) VOR5 and PBO in sSL, (3) VOR10 and PBO in sSE, or (4) VOR5 and PBO in sSE. To control the overall type I error at the 5% significance level, a fixed-sequence approach was applied to these hypotheses in the above-mentioned order. If statistical significance at the *p* < .05 level was not observed in the prior test, subsequent test results were deemed invalid. No multiplicity adjustment was performed on other efficacy analyses.

The sample size was estimated based on the findings from a previous phase 2b study. The LSM differences and their common standard deviation (SD) in sSL at week 2 between VOR5 or VOR10 and PBO were −6.1 ± 16.8 min and −9.3 ± 16.8 min, respectively. For sSE at week 2, the LSM differences and their common SD were 2.32 ± 7.63% and 2.99 ± 7.63%, respectively. To have at least 80% power to detect statistically significant differences on all four primary analyses using a two-sided, two-sample test at the 5% significance level, the sample size was calculated at 171 for each group. Allowing for discontinuation from the study, the planned sample size was set as 180 for each group. An interim analysis was not scheduled.

For responder analysis, three clinically meaningful thresholds were defined: decrease of 20 min or more (i.e., improvement) from baseline at week 2 on sSL, increase of 10% or more from baseline at week 2 for sSE, and increase of 30 min or more from baseline at week 2 for sTST [7].

The safety analysis was performed on the safety analysis set which included all patients who received at least one dose of the investigational drug during the treatment period. The safety analyses in each endpoint were summarized using descriptive statistics. All statistical analyses were performed using SAS version 9.4 (SAS Institute, Tokyo, Japan). No post hoc analyses are included in this report. The dataset is not publicly available due to privacy and proprietary restrictions. Data sharing exemption was approved by the Editor-in-Chief.

## Results

### Disposition, demographics, and baseline characteristics

**Figure 1:**
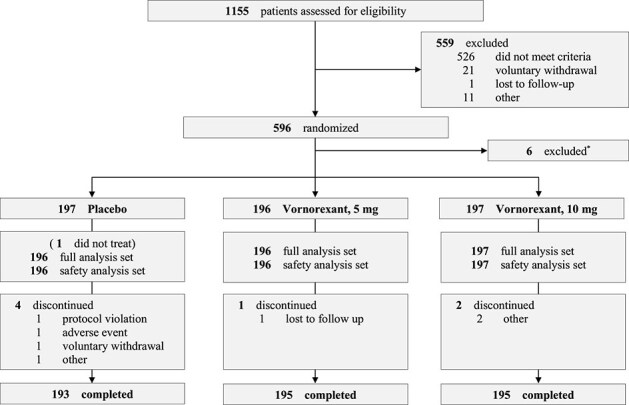
Study flowchart. ^*^Data of the patients from the site committing GCP violation were not included in all analyses.

This study was conducted at 68 investigational sites in Japan, starting in August 2022 and completed in December 2023. One thousand and one hundred fifty-five patients were enrolled during the PBO run-in period, of whom 596 (51.6%) were allocated to one of three groups to double-blind treatment (Figure 1). Among the 596 patients, six were excluded from all analyses prior to the key break due to their participation in a clinical site committing serious GCP violations. Of 590 patients, one in the PBO group did not take any investigational drug; thus, 196, 197, and 196 patients were included in both the full analysis set and safety analysis set in the VOR5, VOR10, and PBO groups, respectively. Most patients completed the study (195 [99.5%] in VOR5, 195 [99.0%] in VOR10, 193 [98.0%] in PBO). A good treatment compliance was observed in this study. Most patients, except three in the PBO group and one in the 5 mg group, had a treatment compliance rate of ≥80%. Furthermore, no significant concomitant medications affecting efficacy or safety endpoints were administered. The patient demographics and baseline characteristics were balanced in all three groups (Table 1).

**Table 1:** Demographics and baseline characteristics (full analysis set)

	PBO (*n* = 196)	VOR 5 mg (*n* = 196)	VOR 10 mg (*n* = 197)
**Sex, *n* (%)**			
Male	94 (48.0)	94 (48.0)	81 (41.1)
Female	102 (52.0)	102 (52.0)	116 (58.9)
**Age, years**			
Mean (SD)	53.4 (12.8)	54.0 (13.1)	52.9 (12.8)
<65, *n* (%)	152 (77.6)	154 (78.6)	154 (78.2)
≥65, *n* (%)	44 (22.4)	42 (21.4)	43 (21.8)
**Body mass index, kg/m** ^**2**^			
Mean (SD)	22.36 (3.50)	22.81 (3.58)	22.67 (3.76)
<25.0, *n* (%)	149 (76.0)	142 (72.4)	148 (75.1)
≥25.0, *n* (%)	47 (24.0)	54 (27.6)	49 (24.9)
**Duration of disease, years**			
Mean (SD)	7.03 (8.19)	6.38 (6.89)	6.28 (6.78)
Median	4.50	4.00	3.80
**Sleep diary, upper: mean (SD), lower: min, max**			
sSL, min	58.9 (28.8) 26, 194	57.0 (26.4) 22, 190	58.6 (28.0) 26, 231
sSE, %	72.98 (11.09) 27.8, 91.3	74.19 (9.18) 39.2, 90.2	73.30 (9.77) 37.0, 89.0
sTST, min	325.1 (49.5) 122, 400	332.9 (41.0) 153, 396	328.4 (42.6) 183, 408
sWASO, min	62.9 (40.0) 0, 257	60.0 (34.5) 0, 189	62.4 (36.4) 0, 201
**ISI total score**			
Mean (SD)	18.9 (2.7)	18.8 (2.6)	19.3 (2.9)

### Primary and key secondary efficacy endpoints

**Figure 2:**
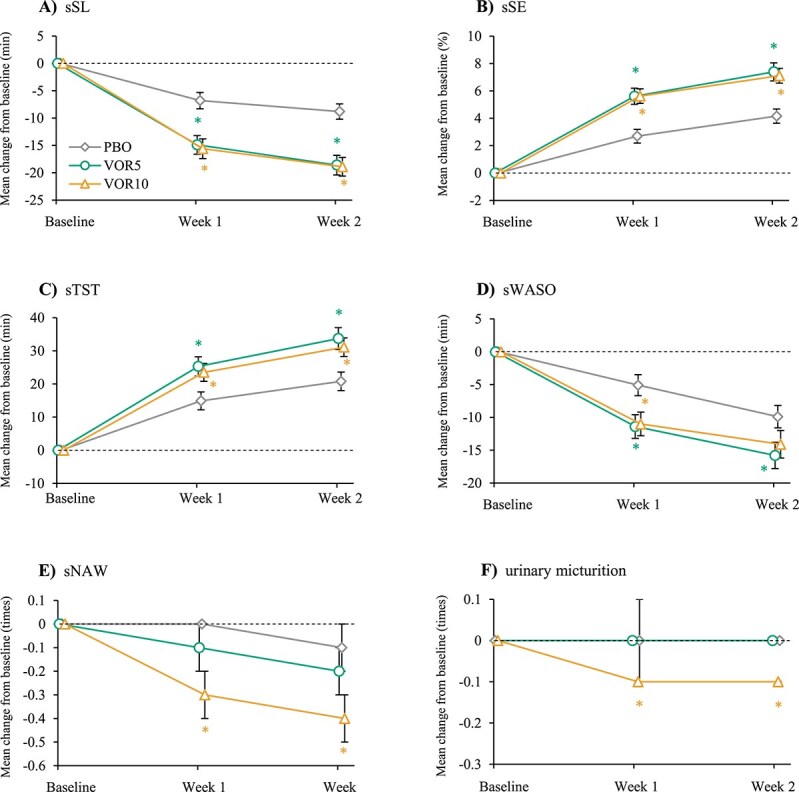
Mean changes from baseline in sleep diary endpoints. (A) sSL (primary endpoint). (B) sSE (key secondary endpoint). (C) sTST (secondary endpoint). (D) sWASO (secondary endpoint). (E) sNAW (secondary endpoint). (F) Urinary frequency (secondary endpoint). Error bars show SEM. Diamond: PBO, circle: VOR5, triangle: VOR10. ^*^p < .05 vs PBO.

The efficacies of the primary (sSL) and key secondary (sSE) endpoints are summarized in Figure 2 and Table 2. The mean decrease in sSL from baseline in VOR10 at week 2 was significantly larger than that in PBO (LSM difference from PBO, −10.1 min; 95% CI = −13.8% to −6.5%; *p* < .001). Additionally, the mean decrease in sSL from baseline in VOR5 at week 2 was also significantly larger than that in PBO (LSM difference from PBO, −10.6 min; 95% CI = −14.2% to −7.0%; *p* < .001).

**Table 2:** Primary and key secondary efficacy endpoints (full analysis set)

	PBO (*n* = 196)	VOR 5 mg (*n* = 196)	VOR 10 mg (*n* = 197)
**Primary endpoint**			
**sSL at week 1, min**			
LSM change from baseline (95% CI)	−6.5 (−9.0, −3.9)	−15.6 (−18.1, −13.0)	−15.4 (−17.9, −12.8)
LSM difference from placebo (95% CI)		−9.1 (−12.7, −5.5)	−8.9 (−12.5, −5.3)
*p*-value		<.001	<.001
**sSL at week 2, min**			
LSM change from baseline (95% CI)	−8.6 (−11.1, −6.0)	−19.2 (−21.7, −16.6)	−18.7 (−21.3, −16.2)
LSM difference from placebo (95% CI)		−10.6 (−14.2, −7.0)	−10.1 (−13.8, −6.5)
*p*-value		<.001	<.001
**Key secondary endpoint**			
**sSE at week 1, %**			
LSM change from baseline (95% CI)	2.58 (1.56, 3.59)	5.77 (4.76, 6.79)	5.58 (4.57, 6.59)
LSM difference from placebo (95% CI)		3.19 (1.75, 4.63)	3.00 (1.57, 4.44)
*p*-value		<.001	<.001
**sSE at week 2, %**			
LSM change from baseline (95% CI)	4.13 (3.04, 5.23)	7.55 (6.46, 8.64)	7.07 (5.99, 8.16)
LSM difference from placebo (95% CI)		3.41 (1.87, 4.96)	2.94 (1.40, 4.48)
*p*-value		<.001	<.001

The values for each evaluation item in the sleep diary represent the average over 7 days. Changes from baseline values at weeks 1 and 2 were analyzed using a linear MMRM, with treatment, period, and treatment-by-period interaction as fixed effects, and baseline values as a covariate. This analysis assumed that missing data were MAR and no imputation was performed.

The mean increase in sSE from baseline in VOR10 was significantly larger than that in PBO (LSM difference from PBO, 2.94%; 95% CI = 1.40% to 4.48%; *p* < .001). In addition, the mean increase in sSE from baseline in VOR5 was also significantly larger than that in PBO (LSM difference from PBO, 3.41%; 95% CI = 1.87% to 4.96%; *p* < .001).

These significant effects in the primary and key secondary endpoints were also observed at an earlier timepoint. At week 1, the changes in sSL and sSE from baseline in both VOR5 and VOR10 were significantly larger than those in PBO (all comparison, *p* < .001) (Figure 2; Table 2). These initial changes in sSL at week 1 were slightly increased at week 2, but the changes in sSE were almost comparable.

**Figure 3:**
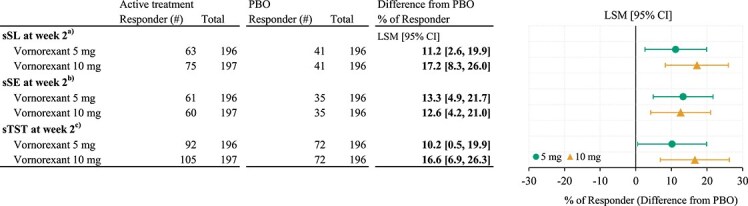
Responder analysis based on sleep diary measurement. The number of patients whose treatment effect was larger than the clinically meaningful threshold. Data are presented as point estimate of the difference from PBO with 95% CIs. a) Patients whose sSL improved from baseline by at least 20 minutes. b) Patients whose sSE improved from baseline by at least 10%. c) Patients whose sTST improved from baseline by at least 30 min.

The numbers of responders in sSL increased with dose increment (63 [32.1%] in VOR5, 75 [38.1%] in VOR10, and 41 [20.9%] in PBO, respectively), and these 95% CIs of differences from PBO in both doses did not include 0 (VOR5: LSM difference from PBO, 11.2%; 95% CI = 2.6% to 19.9%. VOR10: LSM difference from PBO, 17.2%; 95% CI = 8.3% to 26.0%). In sSE, there were more responders in VOR5 and VOR10 than in PBO (61 [31.1%] in VOR5, 60 [30.5%] in VOR10, and 35 [17.9%] in PBO, respectively), and these 95% CIs of differences from PBO in both doses also did not include 0 (VOR5: LSM difference from PBO, 13.3%; 95% CI = 4.9% to 21.7%. VOR10: LSM difference from PBO, 12.6%; 95% CI = 4.2% to 21.0%) (Figure 3).

### Secondary efficacy endpoints

The efficacies assessed by the sleep diary other than the primary and key secondary endpoints are described in Table 3 and Figure 2. The increases in sTST from baseline for VOR5 and VOR10 were significantly larger than that in PBO in both weeks 1 and 2. Additionally, the responders for sTST increased with dosage, and the 95% CI of differences from PBO for both doses did not include 0 (Figure 3). In a similar manner, the decreases in sWASO from baseline for VOR5 and VOR10 were significantly larger than that in PBO in both weeks 1 and 2 except VOR10 at week 2 (Table 3; Figure 2). In sNAW and the frequency of nighttime urination, the decreases from baseline for VOR10 were significantly larger than those in PBO in both weeks 1 and 2, but the change from baseline for VOR5 were comparable to PBO in both weeks (Table 3; Figure 2).

**Table 3:** Secondary efficacy endpoints (full analysis set)

	PBO (*n* = 196)	VOR 5 mg (*n* = 196)	VOR 10 mg (*n* = 197)
**sTST at week 1, min**			
LSM change from baseline (95% CI)	14.1 (8.8, 19.4)	26.2 (20.9, 31.4)	23.4 (18.1, 28.6)
LSM difference from placebo (95% CI)		12.1 (4.6, 19.6)	9.3 (1.9, 16.8)
*p*-value		.002	.014
**sTST at week 2, min**			
LSM change from baseline (95% CI)	20.4 (14.7, 26.1)	34.5 (28.9, 40.2)	31.0 (25.4, 36.6)
LSM difference from placebo (95% CI)		14.1 (6.1, 22.2)	10.6 (2.6, 18.6)
*p*-value		.001	.009
**sWASO at week 1, min**			
LSM change from baseline (95% CI)	−4.8 (−8.1, −1.6)	−11.8 (−15.1, −8.5)	−10.9 (−14.2, −7.7)
LSM difference from placebo (95% CI)		−7.0 (−11.6, −2.4)	−6.1 (−10.7, −1.5)
*p*-value		.003	.010
**sWASO at week 2, min**			
LSM change from baseline (95% CI)	−9.9 (−13.4, −6.3)	−16.2 (−19.8, −12.6)	−14.0 (−17.5, −10.4)
LSM difference from placebo (95% CI)		−6.3 (−11.4, −1.3)	−4.1 (−9.2, 0.9)
*p*-value		.014	.109
**sNAW at week 1, times**			
LSM change from baseline (95% CI)	0.0 (−0.2, 0.1)	−0.2 (−0.3, 0.0)	−0.3 (−0.4, −0.2)
LSM difference from placebo (95% CI)		−0.1 (−0.3, 0.0)	−0.3 (−0.4, −0.1)
*p*-value		.163	.001
**sNAW at week 2, times**			
LSM change from baseline (95% CI)	−0.1 (−0.2, 0.0)	−0.2 (−0.3, −0.1)	−0.3 (−0.5, −0.2)
LSM difference from placebo (95% CI)		−0.1 (−0.3, 0.0)	−0.3 (−0.4, −0.1)
*p*-value		.051	.001
**Urinary frequency at week 1, times**			
LSM change from baseline (95% CI)	0.1 (0.0, 0.1)	0.0 (−0.1, 0.0)	−0.1 (−0.1, 0.0)
LSM difference from placebo (95% CI)		−0.1 (−0.2, 0.0)	−0.1 (−0.2, 0.0)
*p*-value		.114	.021
**Urinary frequency at week 2, times**			
LSM change from baseline (95% CI)	0.0 (−0.1, 0.1)	0.0 (−0.1, 0.0)	−0.1 (−0.1, 0.0)
LSM difference from placebo (95% CI)		0.0 (−0.1, 0.0)	−0.1 (−0.2, 0.0)
*p*-value		.320	.046
**CGI-I at week 1**			
LSM value (95% CI)	3.5 (3.4, 3.6)	3.1 (3.0, 3.2)	3.1 (3.0, 3.3)
LSM Difference from placebo (95% CI)		−0.4 (−0.5, −0.2)	−0.3 (−0.5, −0.2)
*p*-value		<.001	<.001
**CGI-I at week 2**			
LSM value (95% CI)	3.4 (3.2, 3.5)	2.9 (2.8, 3.1)	3.0 (2.9, 3.1)
LSM difference from placebo (95% CI)		−0.4 (−0.6, −0.3)	−0.3 (−0.5, −0.2)
*p*-value		<.001	<.001
**CGI-S at week 1**			
LSM change from baseline (95% CI)	−0.2 (−0.3, −0.1)	−0.5 (−0.6, −0.4)	−0.5 (−0.6, −0.4)
LSM difference from placebo (95% CI)		−0.3 (−0.5, −0.2)	−0.3 (−0.5, −0.2)
*p*-value		<.001	<.001
**CGI-S at week 2**			
LSM change from baseline (95% CI)	−0.3 (−0.5, −0.2)	−0.7 (−0.9, −0.6)	−0.7 (−0.8, −0.5)
LSM difference from placebo (95% CI)		−0.4 (−0.6, −0.2)	−0.3 (−0.5, −0.1)
*p*-value		<.001	<.001

The values for each evaluation item in the sleep diary represent the average over 7 days. Changes from baseline values at weeks 1 and 2 were analyzed using a linear MMRM, with treatment, period, and treatment-by-period interaction as fixed effects, and baseline values as a covariate. This analysis assumed that missing data were MAR and no imputation was performed. No adjustment for multiplicity was performed.

In parallel with the patient-reported outcomes (sleep diary), the clinician-rated scores (CGI-S and CGI-I) also showed significant improvements over PBO at both doses and timepoints (Table 3).

### Safety

AEs during the treatment periods (2-week double-blind period, and 1-week PBO run-out period) are summarized in Table 4. Patients who received VOR5, VOR10, or PBO had a similar incidence of AEs. No death or serious AEs were reported throughout the study. All AEs were mild or moderate in severity. The most common (≥3%) AEs with VOR5 and VOR10 were somnolence and nasopharyngitis, but all incidences were mild in severity. No AEs of cataplexy, falls, muscular weakness, sleep paralysis, hypnagogic or hypnopompic hallucinations, drug abuse or excessive daytime sleepiness were reported. No events of ECIs for dependency, withdrawal symptoms, rebound insomnia, or suicidal ideation/behavior were observed during the entire study.

**Table 4:** AEs during treatment period (safety analysis set)

	PBO (*n* = 196)	VOR 5 mg (*n* = 196)	VOR 10 mg (*n* = 197)
**AE, *n* (%)**			
≥1 adverse event	25 (12.8)	29 (14.8)	32 (16.2)
≥1 treatment-related AE	4 (2.0)	10 (5.1)	13 (6.6)
Leading to death	0	0	0
Serious except death	0	0	0
Severe	0	0	0
Leading to discontinuation	1 (0.5)	0	0
Leading to suspension of study drug	0	1 (0.5)	0
**Participants with AE (≥1% in any group)**			
Somnolence	3 (1.5)	6 (3.1)	7 (3.6)
Nasopharyngitis	3 (1.5)	1 (0.5)	6 (3.0)
Headache	2 (1.0)	4 (2.0)	2 (1.0)
Blood creatine phosphokinase increased	1 (0.5)	2 (1.0)	4 (2.0)
Blood triglycerides increased	2 (1.0)	1 (0.5)	0
C-reactive protein increased	1 (0.5)	2 (1.0)	0
Aspartate aminotransferase increased	0	0	2 (1.0)
Protein urine present	0	0	2 (1.0)
Arthralgia	2 (1.0)	0	0
Myalgia	2 (1.0)	0	0
**ECIs**			
Residual effects	3 (1.5)	6 (3.1)	8 (4.1)
Related to dependency	0	0	0
Related to withdrawal symptoms	0	0	0
Related to rebound insomnia	0	0	0
Related to suicidality	0	0	0

Data are shown as *n* (%). AEs that occurred after the first dose during the treatment period in the safety analysis set were summarized with preferred terms of MedDRA ver. 26.1. Definitions of each ECI are shown in Supplementary Material S1.

KSS scores and changes from baseline obtained at each visit are summarized in Table 5. At both week 1 and week 2, no score increments were observed in all groups compared to baseline. Furthermore, none of VOR groups exhibited higher scores than PBO at any time point.

**Table 5:** Next-day residual effect

Endpoint	PBO	VOR 5 mg	VOR 10 mg
**KSS**			
Baseline^*^	5.2 (2.2)	4.9 (2.1)	5.1 (2.2)
End of the DB-treatment^†^	4.7 (2.1)	4.1 (2.1)	4.7 (2.1)
Change from baseline^‡^	−0.4 (1.9)	−0.7 (1.8)	−0.4 (1.9)

Data are shown in mean (SD).

^*^Sample sizes were 196 for PBO, 196 for VOR 5 mg, 197 for VOR 10 mg.

^†^Sample sizes were 195 for PBO, 196 for VOR 5 mg, 197 for VOR 10 mg.

^‡^Sample sizes were 195 for PBO, 196 for VOR 5 mg, 197 for VOR 10 mg.

No remarkable changes from baseline in laboratory tests, vital signs, or body weight were observed among the treatment groups. Abnormal electrocardiography findings were not found in any patient. No evidence of suicidality was shown by C-SSRS assessment. Assessment of rebound insomnia is shown in Supplementary Material S1. The mean values of sSL, sSE, sTST, and sWASO did not worsen from baseline in the average of the first three nights during the PBO run-out. Withdrawal symptoms did not develop in any treatment group; the 20-item total scores of BWSQ decreased (i.e., not worsened) during the PBO run-out period compared to baseline, and these decreases were comparable among all treatment groups (Supplementary Material S1). A single case of intentional drug misuse was identified in VOR10 only, involving an additional 10 mg administration during daytime on Day 13.

## Discussion

This phase 3 clinical study was designed to examine the efficacy and safety of the dual OX_1/2_ receptor antagonist, vornorexant, in nonelderly and elderly patients with insomnia. The study met both the primary and key secondary objectives, confirming that treatment with vornorexant improved subjective measures of sleep onset latency and sleep efficiency more effectively than PBO. These effects were observed at both doses (5 and 10 mg) and both timepoints (weeks 1 and 2). Vornorexant generally showed significant improvement in the physician-rated insomnia severity as well as the sleep diary. Vornorexant was well tolerated, safe, and showed no narcolepsy-related signs.

Difficulty in initiating sleep is a pervasive symptom of insomnia. Its prevalence was reported to be greater than difficulty maintaining sleep or early morning awakening with difficulty resuming sleep across all age categories [40]. As shown in Figure 2 and Table 2, vornorexant significantly ameliorated difficulty in initiating sleep from week 1 and the effect was maintained at least to week 2, indicating its rapid onset of action without evidence of reduced effects over time. Existence of sleep-inducing effects of vornorexant was demonstrated not only by the “mean-wise” analysis but also by the responder analysis, reinforcing the existence of the sleep-inducing effect of vornorexant. These results were consistent with the rapid absorption of vornorexant after oral administration [25, 26].

The sleep maintenance effect of vornorexant could be considered from multiple perspectives using sleep diary outcomes (sSE, sTST, and sWASO) assessed at various doses and timepoints. Of these, all results except sWASO at week 2 with 10 mg vornorexant demonstrated significant improvement. Three potential factors should be considered when interpreting the results. The first is that inter-individual variability for recognition of sWASO may have been larger than that of the other parameters. The mean baseline values of sWASO ranged from 60.0 to 62.9 min across all groups (Table 1). However, patients with an sWASO of 0 min at baseline (which could not be improved any further) were observed in each group. This is likely due to the absence of exclusion criteria based on sWASO during the PBO run-in period of this study. The second is that improvements in sSE and sTST may theoretically result from reductions in sSL, even in the absence of changes in sWASO. The third factor is that the observed improvements in sSE, sTST, and sWASO may have been smaller than the thresholds for clinical significance as defined by the American Academy of Sleep Medicine clinical practice guidelines [7]. Among these, the second and third factors may be attributable to the relatively short half-life of vornorexant.

The efficacy of 5- and 10-mg doses was found to be mostly comparable in this study. Meanwhile, some results from the responder analysis indicated a dose-related effect. No noticeable biases in demographic or baseline parameters were observed among groups. Phase 1 pharmacokinetic studies of vornorexant indicated that the dose strengths used in this study were within the range for dose proportionality in terms of both Cmax and area under the curve [25]. Furthermore, the pharmacodynamic outcomes up to 4 h post-administration also showed dose response without evident ceiling effects up to 30 mg [25]. From these, although the reason that the mean-wise analysis did not provide a clear dose-related effect remains unclear, the proportion of responders increased with the dose, suggesting that increasing the dosage of vornorexant may be beneficial for some patients in a clinical setting.

In this study, a small but significant reduction in nocturnal micturition was detected with high-dose (10 mg) of vornorexant treatment. This finding should be noted because no previous confirmatory phase 3 studies have reported such findings with DORAs to the best of our knowledge. Together with our results, anatomical [41], pharmacological [42], and clinical evidence [43, 44] may support the hypothesis that DORAs reduce nocturnal micturition through their mechanism of action. The significant reduction in sNAW observed with the same dose (10 mg) of vornorexant might partly reflect decreased nocturnal voiding. Indeed, over half of those complaining of poor sleep reported that about half of their nightly awakenings were associated with nocturnal voiding [45]. Although the causal relationship between reduced nocturnal micturition and decreased sNAW remains unclear, these effects may offer additional benefits in treating insomnia, particularly in elderly patients [46].

In the evaluation of daytime sleepiness using the KSS, scores at week 1 and week 2 did not increase compared to PBO. This result may be attributable to two factors: the alleviation of sleep debt resulting from improved insomnia symptoms, and the pharmacokinetic profile of vornorexant characterized by a short half-life. While the reduction of sleep debt is also expected with other existing DORAs, the minimal impact on daytime sleepiness due to low residual drug concentrations in the morning may not be as pronounced as with vornorexant.

Both doses of vornorexant were well tolerated in this study, and no patient was discontinued due to any AE in the vornorexant groups. The incidence of somnolence was low in both vornorexant groups and was mild in severity. No AEs of falls, narcolepsy, cataplexy, sleepwalking, or abuse potentials and dependency were reported. Furthermore, safety outcome measures, including rebound insomnia (sleep diary), BWSQ, and suicidality (C-SSRS) were comparable to PBO. A potential risk of drug abuse liability may be a concern due to the pharmacokinetic characteristics of vornorexant which include rapid absorption and elimination. During the study, only one case of intentional drug misuse was observed in VOR10 and no abuse-related AEs were observed. These findings suggest that vornorexant is unlikely to have significant abuse potential. From these data, no noteworthy safety or tolerability concerns of vornorexant were observed. However, a definitive assessment of the abuse liability of vornorexant should be supported by a human abuse liability study, as generally recommended by the FDA [47].

Several points limit the interpretation of these results. Patients in this study were Japanese only, and so the observations in this study may not be directly extrapolated to other races or ethnicities. In addition, despite having an agreement with regulatory authorities, the treatment period was short and patients with psychiatric disorders were not enrolled. Effects on patients with more comprehensive backgrounds, including psychiatric disorders, should be considered in a separate long-term study. Finally, objective sleep measurements using PSG or actigraphy were not performed in this study. Thus, the sleep values presented in this report should be interpreted with slightly caution, taking into account the potential inconsistency between objective and subjective measures. A meta-analysis comparing actigraphy and sleep diaries in patients with insomnia reported that both methods yielded comparable treatment responses across key sleep parameters (sSL, sSE, sTST, and sWASO) [48]. This suggests that the sleep diaries alone may provide sufficiently reliable data. However, these results should not be compared with other pharmacological intervention studies that screened patients with PSG parameters.

## Conclusion

This study confirmed the treatment effects of vornorexant compared to PBO, based on subjective measurements of sleep latency and sleep efficiency in sleep diaries in Japanese patients with insomnia. These effects were evident at both 5- and 10-mg doses, and at both weeks 1 and 2. It should be noted that the secondary endpoint sWASO at week 2 in the 10-mg group did not reach statistical significance. Vornorexant was generally tolerated with no serious AEs and no evidence of withdrawal symptoms or rebound insomnia.

## Supplementary Material

Supplemental_materials_zsaf291

## Data Availability

Aggregated data supporting the findings of this study are presented in the manuscript and supplementary materials. However, individual patient data are not publicly available due to privacy concerns and proprietary restrictions related to company-owned data. An exemption from data sharing was approved by the Editor-in-Chief.
