# Supplementary material for: Efficacy and safety of vornorexant in Japanese patients with insomnia: a randomized, placebo-controlled phase 3 pivotal study
Source: Sleep. 2025 Sep 26;49(3):zsaf291. doi: 10.1093/sleep/zsaf291 (PMC13017578; doi:10.1093/sleep/zsaf291)
Supplement: Supplemental_materials_zsaf291 [file supplemental_materials_zsaf291.pdf]

Efficacy and safety of vornorexant in Japanese patients with insomnia: A randomized, placebo-controlled phase 3 pivotal study

Makoto Uchiyama,[a,b*] Daiji Kambe,[c] Sayaka Hasegawa,[c] Yumiko Imadera,[c] Hironori Yamasaki,[c] Naohisa Uchimura[d]

[a]Tokyoadachi Hospital, 5-23-20 Hokima, Adachi, Tokyo 121-0064, Japan

[b]Department of Psychiatry, Nihon University School of Medicine, Oyaguchi-Kamicho, Itabashi 173-8610 Tokyo, Japan

[c]Development Headquarters, Taisho Pharmaceutical Co., Ltd., 3-24-1 Takada, Toshima, Tokyo 170-8633, Japan

[d]Department of Neuropsychiatry, Kurume University School of Medicine, 67 Asahi-machi, Kurume, Fukuoka 830-0011, Japan

*** Corresponding author**
Makoto Uchiyama
Tokyoadachi Hospital, 5-23-20 Hokima, Adachi, Tokyo 121-0064, Japan
Tel: +81-3-3883-6331
E-mail: maco.uchiyama@nifty.com

Material S1

A full list of the clinical study sites of this study

Ai Sakura Clinic

Aoba Mental Clinic

Daido Clinic

Fukuoka Urasoe Clinic

Fukuwa Clinic

Gokeikai Osaka Kaisei Hospital

HIGASHI-SAPPORO MENTAL CLINIC

Hillside Clinic Jingumae

Institute Medical Corporation Hitomikai Motomachi Takatsuka Naika Clinic

Itakura Clinic

Kawamura Mental Clinic

Keisen second clinic

Kimura Medical Clinic

Kinugawa Cardiology Clinic

Koishikawa Tokyo Hospital

Kokoro no Clinic Hirao

Komagamine clinic

Kouwa Clinic

Kuwamizu Hospital

Kyotani clinic

Kyowakai Healthcare Corporation Hannan Hospital

Mabashi Clinic

Maebashi Hirosegawa Clinic

Medical Corporation Asbo Tokyo Asbo Clinic

Medical Corporation Heishinkai OCROM Clinic

Medical Corporation Heishinkai ToCROM Clinic

Medical corporation Shinanokai Samoncho Clinic

Medical corporation Shirayurikai Swing Nozaki Clinic

Medical corporation Yuhokai Miho Clinic

Meguro Station East Exit Mental Clinic

Meiwa Hospital

Mental Clinic Sakurazaka

Minami-Aoyama Antique Street Clinic

Minna no Suimin Stress Care Clinic

Mito Mental Clinic

Miyazawa Clinic

NAKANO ekimae mental clinic

National Center of Neurology and Psychiatry

Nishikasai ekimae Family Clinic

Ota Sleep Disorders Center

Rainbow & Sea Hospital

RESM respiratory and sleep medical-care clinic

Saino Clinic

Sakurazaka Clinic SophyAnce

Sangenjaya Nakamura Mental Clinic

Sangubashi Kokorono clinic

Sapporo Hanazono Hospital

Sapporo Yuushoukan Hospital

Sasaki Clinic

Sekino Hospital

Shibasaki Internal medicine & Pediatrics Clinic

Shibuya Shin-minamiguchi Clinic

Shimode Mental Clinic

Sleep & Stress Clinic

Sleep Clinic Chofu

Sleep Support Clinic

Sugiura clinic

Suzuki Internal & Circulatory Medical Clinic

Tai Clinic

Takeuchi Hospital

Tatsuta Clinic

Toda Internal medicine & Neurology Clinic

Tokyo-Eki Center-building Clinic

Touei Internal Medicine Clinic

Yamatenomori kokorono Clinic

Yotsuya Internal Medicine

You Ariyoshi Sleep Clinic

Yoyogi Sleep Disorder Center

Material S2

Inclusion criteria

Those who met all the following criteria were included in this study.

(1) Japanese male and female who are aged 18 years or older at the time of informed consent
(2) Outpatients
(3) Patients diagnosed in insomnia disorder by the Diagnostic and Statistical Manual of Mental Disorders, 5th edition (DSM-5)
(4) Patients with Insomnia Severity Index (ISI) score ≥15 at both Visit 1 and Visit 3
(5) Patients with persistent sleep difficulty for 2 weeks just before Visit 1
   · Sleep latency ≥ 30 min on at least 3 nights per week
   · Wake time after sleep onset ≥ 30 min on at least 3 nights per week
(6) Patients who habitually spend 6.5–9 h in bed
(7) Patients who habitually go to bed between 21:00 and 1:00
(8) Patients who adhere to an administration regimen of investigational drug ≥80% from Visit 2 to Visit 3
(9) Patients whose sleep history meets all the following criteria, as recorded in the sleep diary for the 7 days immediately preceding Visit 3
   · Completion of the sleep diary for more than 6 nights (if any item is missing on a given night, that night is not considered complete)
   · Sleep latency (sSL) ≥ 30 min per night on ≥ 4 days
   · Total sleep time (sTST) < 6.5 h per night on ≥ 4 days
   · Time spent ≥ 6.5 to ≤ 9 h in bed per night on ≥ 4 days
   · Going to bed between 21:00 and 1:00 on ≥ 4 days
(10) Patients who understand how to use the electronic patient-reported outcome diary (ePRO) and are willing to record their own sleep data using the ePRO throughout the study period
(11) Patients who are willing to adhere to appropriate contraceptive methods (including contraception for both the patient and their partner: the use of condoms, intrauterine devices, or pessaries) from the time of obtaining informed consent until Visit 6 (or until the discontinuation of the study)
(12) Patients who understand the study details, and provide their written informed consent

Exclusion criteria

Those who met any of the following criteria were excluded from this study.

(1) Patients diagnosed in any disorder (except insomnia disorder) among sleep-wake disorders by the DSM-5
(2) Patients with the score of the Japanese version of STOP-Bang test ≥ 5 at Visit 1
(3) Patients who showed a reduction of more than 5 points in the ISI score at Visit 3 compared to Visit 1
(4) Patients experiencing sleep difficulties due to their medical conditions, including pain, pruritus, hot flashes, nocturia (more than 3 times per night), cardiovascular disease, bronchial asthma, reflux esophagitis, endocrine disease, and periodic limb movement disorder
(5) Patients with psychiatric diseases, including depression, schizophrenia, and anxiety neurosis
(6) Patients who have exhibited suicidal ideation or any suicidal behavior, as determined by the Columbia-Suicide Severity Rating Scale (C-SSRS) assessed at Visits 1 or 3, or patients who have a history of suicidal ideation or suicide attempts within the past 5 years.
(7) Patients who have habitual behavior that influence their own sleep including daily nap and using smartphone or TV in

bed)

(8) Patients who have traveled across 3 or more time zones within 4 weeks before Visit 1, or those who plan to travel abroad during the study

(9) Patients who have experienced any irregular or night shift work within 4 weeks before Visit 1 or those who scheduled similar work during the study

(10) Patients with organic brain diseases, including neurodegenerative or cerebrovascular disorders, or epilepsy

(11) Patients who meet any of the following criteria in terms of blood pressure or pulse rate measured while seated at Visit 1.

- Systolic blood pressure > 160 mmHg
- Diastolic blood pressure > 100 mmHg
- Pulse rate > 100 bpm

（Even if initial measurements meet the criteria, the study may continue if a re-measurement, at the investigator's discretion, does not meet the criteria. A re-measurement can be done once on the same day）

(12) Patients with a history of poorly controlled diabetes mellitus (hemoglobin A1c [HbA1c] > 8%) within 6 months before Visit 1

(13) Patients who meet any of the following criteria in terms of laboratory values at Visit 1

- Alanine aminotransferase (ALT) level > 2.5 times the upper limit of the reference value
- Aspartate aminotransferase (AST) level > 2.5 times the upper limit of the reference value
- Total bilirubin level > 1.5 times the upper limit of the reference value

(14) Patients with any serious comorbidities (severity were identified with reference to Grade 3 in Common Terminology Criteria for Adverse Events version 5.0

(15) Patients who are or have a history of malignant tumors (Patients are eligible for this study if they have been in remission for over 5 years without any medical treatment and show no signs of recurrence)

(16) Patients with a history of drug allergy

(17) Patients with serious allergic predisposition, including asthma requiring treatment

(18) Patients who are or have a history of drug abuse, drug dependence, or alcohol dependence

(19) Patients who are judged by the principal investigator or sub-investigator to be inappropriate for participation in this study due to any abnormalities in vital signs, standard 12-lead electrocardiogram, or laboratory values at Visits 1 to 3

(20) Patients who test positive in the urine drug test at Visit 1

(21) Patients who have used or are scheduled to use prohibited concomitant medications

(22) Patients who have modified or are scheduled to be modified restricted concomitant medications (definition of restriction is that changes in dosage, new use, discontinuation, and as-needed use are not permitted)

(23) Patients who have modified or are scheduled to be modified restricted concomitant therapies

(24) Patients who currently participate in any other clinical trial or who have been administered other investigational drugs within 12 weeks before Visit 1

(25) Patients who have been administered vornorexant in any previous study

(26) Women who are pregnant, lactating, potentially pregnant, planning to conceive during the study, or have tested positive for pregnancy during the observation period. Men whose partner is planning to conceive during the study.

(27) Patients who are unwilling to adhere to the clinical guidelines of subject management

(28) Patients who are judged to be inappropriate for participation in this study at any other investigator's discretion

Figure S1

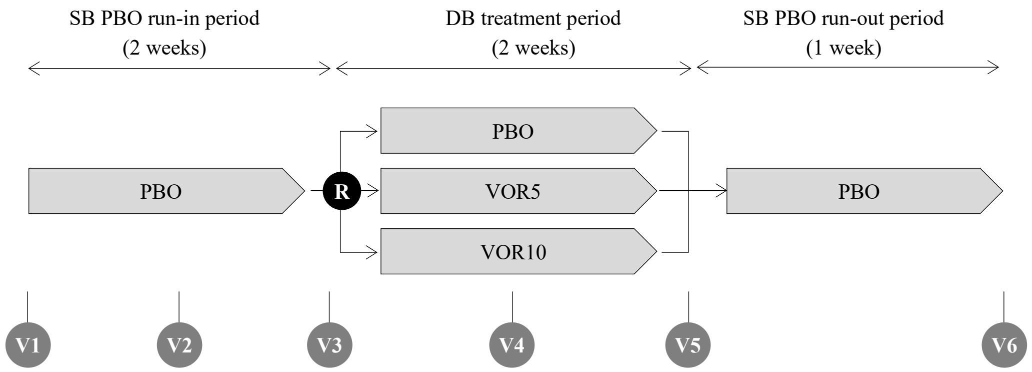

Study design.

Abbreviations: DB, double-blind; PBO, placebo; R, randomized; SB, single-blind; $Vn_{(n=1\sim6)}$, Visit $n_{(n=1\sim6)}$, VOR5: vornorexant 5 mg, VOR10: vornorexant 10 mg

Table S1

ECI	Hierarchy	Items
Residual effects	PT	Fatigue, Feeling abnormal, Sluggishness, Amnesia, Aphasia, Balance disorder, Cognitive disorder, Coordination abnormal, Depressed level of consciousness, Disturbance in attention, Dysarthria, Lethargy, Memory impairment, Retrograde amnesia, Sedation, Somnolence, Stupor, Bradyphrenia, Distractibility, Mental status changes
Related to dependency	SMQ	Drug abuse and dependence
Related to withdrawal symptoms	SMQ	Drug withdrawal
Related to rebound insomnia	PT	Initial insomnia, Middle insomnia, Terminal insomnia
Related to suicidality	SMQ	Suicide/self-injury

Definition of AEs of ECIs. All terms are in MedDRA ver. 26.1.

Abbreviations: AE, adverse event; ECI, events of clinical interest; MedDRA, Medical Dictionary for Regulatory Activities; PT, preferred term; SMQ, standardized MedDRA queries.

Table S2

Endpoint	PBO	VOR 5 mg	VOR 10 mg
**sSL (min)**			
Baseline [a]	58.9 (28.8)	57.0 (26.4)	58.6 (28.0)
PBO run-out [b]	48.9 (31.3)	48.1 (33.2)	50.7 (33.2)
Change from baseline [b]	−10.2 (25.1)	−9.0 (27.1)	−7.9 (27.4)
**sSE (%)**			
Baseline [a]	72.98 (11.09)	74.19 (9.18)	73.30 (9.77)
PBO run-out [b]	77.58 (12.88)	78.55 (11.58)	76.52 (12.93)
Change from baseline [b]	4.67 (9.15)	4.42 (9.95)	3.24 (9.37)
**sTST (min)**			
Baseline [a]	325.1 (49.5)	332.9 (41.0)	328.4 (42.6)
PBO run-out [b]	348.4 (59.2)	354.5 (59.6)	341.8 (61.2)
Change from baseline [b]	23.7 (44.4)	21.8 (53.1)	13.3 (46.8)
**sWASO (min)**			
Baseline [a]	62.9 (40.0)	60.0 (34.5)	62.4 (36.4)
PBO run-out [b]	52.5 (40.9)	49.2 (36.6)	54.3 (39.7)
Change from baseline [b]	−10.6 (28.2)	−11.1 (30.6)	−8.2 (30.2)

Rebound insomnia assessed by sleep diary. Data are shown as mean (SD).

Abbreviations: PBO, placebo; sNAW, subjective number of awakenings; SD, standard deviation; sSE, subjective sleep efficiency; sSL, subjective sleep latency; sTST, subjective total sleep time; sWASO, subjective wake time after sleep onset; VOR, vornorexant

[a] Sample sizes were 196 for PBO, 196 for VOR 5 mg, 197 for VOR 10 mg.

[b] Sample sizes were 193 for PBO, 195 for VOR 5 mg, 196 for VOR 10 mg.

Table S3

Endpoint	PBO	VOR 5 mg	VOR 10 mg
**BWSQ total score (items 1 to 20)**			
Baseline [a]	1.3 (2.6)	1.3 (3.1)	1.3 (2.9)
End of DB treatment [b]	1.1 (2.3)	1.0 (2.2)	1.0 (2.3)
Change from baseline [b]	−0.2 (2.1)	−0.3 (2.4)	−0.3 (1.8)
PBO run-out [c]	0.9 (2.0)	0.7 (1.8)	0.9 (2.3)
Change from baseline [c]	−0.4 (1.7)	−0.5 (2.5)	−0.4 (2.1)

Withdrawal symptoms. Data are shown as mean (SD).

Abbreviations: BWSQ, Benzodiazepine Withdrawal Symptom Questionnaire; DB, double-blind; PBO, placebo; SD, standard deviation; VOR, vornorexant

[a] Sample sizes were 196 for PBO, 196 for VOR 5 mg, 197 for VOR 10 mg.

[b] Sample sizes were 192 for PBO, 194 for VOR 5 mg, 196 for VOR 10 mg.

[c] Sample sizes were 185 for PBO, 191 for VOR 5 mg, 189 for VOR 10 mg.
